# Characteristics of Patent Ductus Arteriosus in Congenital Rubella Syndrome

**DOI:** 10.1038/s41598-019-52936-6

**Published:** 2019-11-19

**Authors:** Michiko Toizumi, Cam Giang T. Do, Hideki Motomura, Tin N. Do, Hirofumi Fukunaga, Makiko Iijima, Nhan NT. Le, Hung Thanh Nguyen, Hiroyuki Moriuchi, Lay-Myint Yoshida

**Affiliations:** 1 Department of Pediatric Infectious Diseases, Institute of Tropical Medicine, Nagasaki University, Nagasaki, Japan; 2 Department of Global Health, School of Tropical Medicine and Global Health, Nagasaki University, Nagasaki, Japan; 3 Department of Cardiology, Children’s Hospital 1, Ho Chi Minh City, Vietnam; 4 Department of Pediatrics, Nagasaki Medical Center, Omura, Japan; 5 Department of Pediatrics, Nagasaki University Hospital, Nagasaki, Japan; 6 Expanded Programme on Immunization, WHO representative office in Viet Nam, Hanoi, Vietnam; 7 Outreach and International Department, Children’s Hospital 1, Ho Chi Minh City, Vietnam; 8 Graduate School of Biomedical Sciences, Nagasaki University, Nagasaki, Japan

**Keywords:** Cardiology, Infectious diseases

## Abstract

This study investigated the characteristics of congenital rubella syndrome (CRS)-associated cardiac complications, particularly patent ductus arteriosus (PDA). We reviewed the medical records of patients with CRS who were admitted to the Children’s Hospital 1 in Vietnam between December 2010 and December 2012, and patients with CRS who underwent PDA transcatheter occlusion therapy at the cardiology department between December 2009 and December 2015. We compared the characteristics of PDA treated with transcatheter closure between children with CRS (CRS-PDA) and those without CRS (non-CRS-PDA) who underwent PDA transcatheter closure between July 2014 and December 2015. One-hundred-and-eight children with CRS were enrolled. Cardiac defects (99%), cataracts (72%), and hearing impairment (7%) were detected. Fifty CRS-PDA and 290 non-CRS-PDA patients were examined. CRS-PDA patients had smaller median birthweight (p < 0.001), more frequent pulmonary (p < 0.001) and aortic stenosis (p < 0.001), higher main pulmonary artery pressure, and higher aortic pressure in systole/diastole (p < 0.001 for each) than did non-CRS-PDA patients. The proportion of tubular-type PDA was higher in CRS-PDA patients (16%) than in non-CRS-PDA patients (3%) (p = 0.020). Tubular-type PDA was frequently seen in patients with CRS and accompanied by pulmonary/systemic hypertension and pulmonary/aortic stenosis; in these patients, more cautious device selection is needed for transcatheter PDA closure.

## Introduction

Outbreaks of rubella and congenital rubella syndrome (CRS) continue to occur in various countries where a rubella-containing vaccine is not included in the national immunization program, particularly in Africa and Asia^1^. Our previous study in 2009–2010 indicated that 30% of pregnant women in Nha Trang, central Vietnam, were susceptible to rubella infection^2^. In 2011, a large-scale rubella outbreak occurred in Vietnam, followed by the emergence of numerous CRS cases^3^. Sixty-eight percent of patients with CRS had cardiovascular complications, with patent ductus arteriosus (PDA) being the most prevalent^4^. Mortality among children with CRS was highly associated with pulmonary hypertension (PH) due to PDA; however, PH in those with CRS could be overcome with transcatheter PDA closure^4^. While PDA has frequently been associated with CRS, its morphologic and hemodynamic characteristics have not been investigated precisely.

An experienced cardiologist who treated many cases of PDA has empirically recognized that PDA associated with CRS (CRS-PDA) is more difficult to treat with transcatheter occlusion therapy, and the proportion of patients with tubular-type of PDA is higher than overall cases of PDA (Do TN, personal communication)^5^. Patients with CRS-PDA may have difficulty in satisfactorily placing and stabilizing a prosthesis as well as a risk of embolization during release^5^. Masri *et al*. have demonstrated that non-conical PDAs have more significant protrusion of the device into the descending aorta than do conical PDAs^6^.

In this study, we 1) described the clinical and cardiac manifestations of children with CRS and 2) investigated the PDA types and sizes, the device types for transcatheter PDA occlusion, and cardiac complications other than PDA among PDA patients with CRS (CRS-PDA) comparing with those without CRS (non-CRS-PDA).

## Results

### Number of interventions for PDA

A total of 2978 catheterization procedures were conducted at Children’s Hospital 1 (CH1) in Ho Chi Minh City, Vietnam, between 2011 and 2015. Among them, 1599 (53.7%) were transcatheter PDA closure procedures. The proportion of PDA closure procedures among all catheterization procedures was higher in 2011 and 2012 than in other years (Supplemental Table 1). The monthly number of transcatheter PDA occlusion procedures, sorted by birth dates, peaked in October and November 2011 (Supplemental figure). Forty-four patients underwent PDA ligation surgery between 2011 and 2015; 20 of these were born in 2011.

### Characteristics of patients with CRS

**Figure 1:**
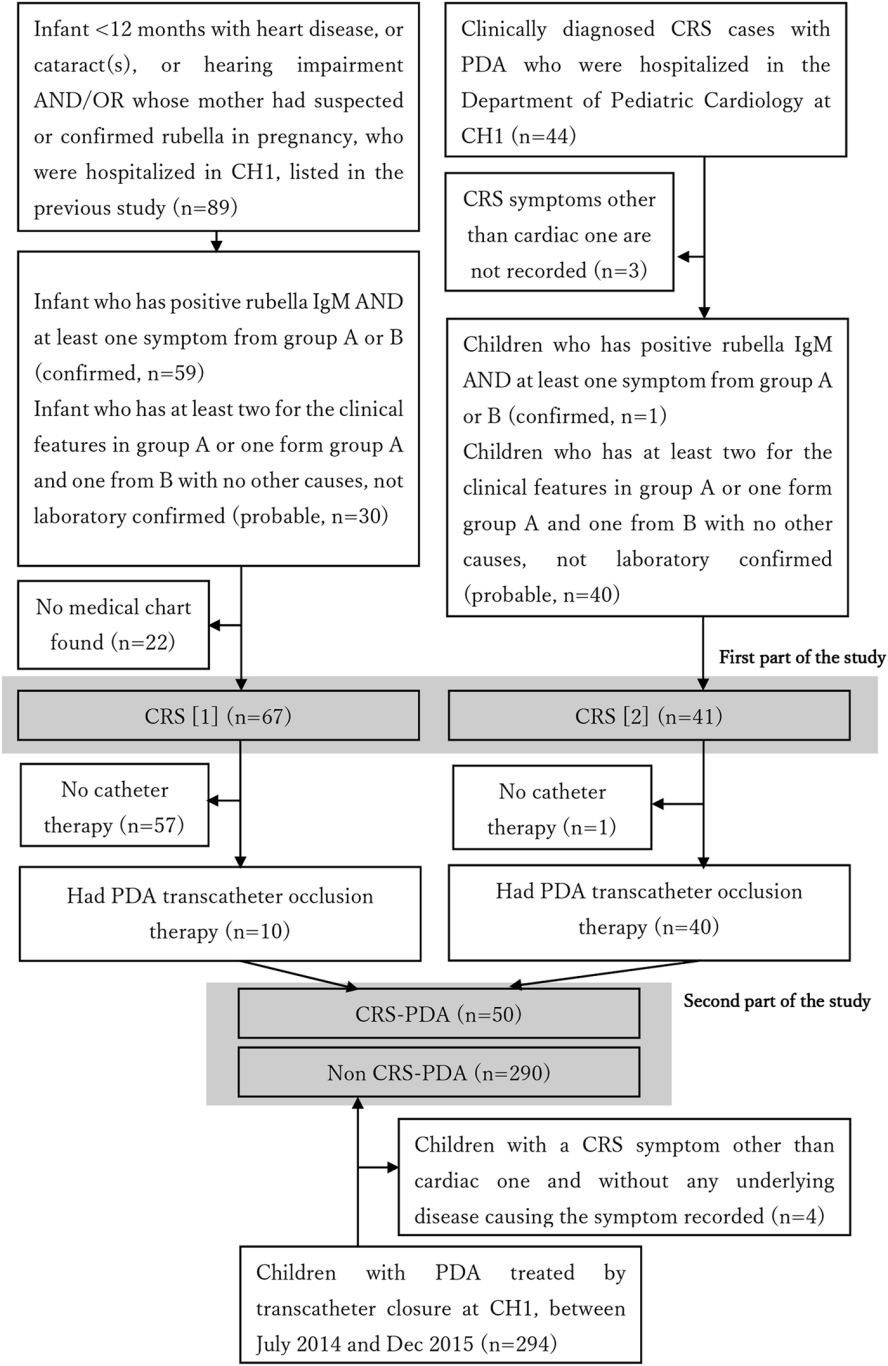
Enrollment flow chart for the study populations. Part 1; a study for characteristics of cases of congenital rubella syndrome, Part 2; a study for patent ductus arteriosus with or without congenital rubella syndrome. Symptoms in Group A; congenital heart disease, cataract(s), glaucoma, and suspected hearing impairment Symptoms in Group B; purpura, jaundice within 24 hours after birth, hepatosplenomegaly, meningoencephalitis, developmental delay, and microcephaly^29^.

We enrolled 67 patients with CRS (46 confirmed/21 probable) admitted between December 2010 and December 2012 who were identified by a previous study^3^, and 41 patients with probable CRS and PDA who were hospitalized and identified from the department’s patient list (Fig. 1). The patient characteristics are shown in Table 1. Echocardiographic studies in children who were actively screened in the previous study^3^ (the middle column in Table 1) showed the proportion of types of cardiac defect in CRS: PDA (79%), tricuspid regurgitation (77%), atrial septal defect (patent foramen ovale) (73%), pulmonary hypertension (50%), mitral regurgitation (29%), pulmonary stenosis (15%), pulmonary regurgitation (14%), and ventricular septal defect (12%). Twenty-five percent of the children from the previous study^3^ died before or soon after discharge.

**Table 1:** Characteristics of children with congenital rubella syndrome.

Characteristics		Total cases of CRS	CRS from previous study^3^	CRS and PDA from the Department list
		Number (%) or median (IQR)^a^	Number (%) or median (IQR)^a^	Number (%) or median (IQR)^a^
		n = 108	n = 67	n = 41
**Demographics**				
Sex (male)		47 (43.5)	30 (44.8)	17 (41.5)
Date of birth (range)		10 Jan 2009–05 Nov 2012	18 Jan 2011–18 Oct 2012	10 Jan 2009–05 Nov 2012
Age on admission (months)		3.3 (0.5–8.3)	1.0 (0.1–3.4)	8.3 (6.2–19.3)
Body weight on admission (kilogram)		3.5 (2.3–5.2)	2.7 (2.1–3.4)	5.4 (4.6–7.1)
**Perinatal information**				
Birthweight (gram)		n = 101	n = 65	n = 36
		2200 (1850–2500)	2100 (1700–2400)	2200 (2000–2600)
Low birthweight (<2500 gram)		73 (72.3)	51 (78.5)	22 (61.1)
		n = 108	n = 67	n = 41
Gestational weeks at birth	37 or more	65 (60.2)	37 (55.2)	28 (68.3)
	less than 37	36 (33.3)	27 (40.3)	9 (22.0)
	Unknown	7 (6.5)	3 (4.5)	4 (9.8)
**Symptoms**				
Cardiac disease	Yes	107 (99.1)	66 (98.5)	41 (100.0)
	No	0 (0.0)	0 (0.0)	0 (0.0)
	Unknown	1 (0.9)	1 (1.5)	0 (0.0)
Symptoms from cardiac disease^a^	Yes	63 (58.3)	28 (41.8)	35 (85.4)
	No	24 (22.2)	20 (29.9)	4 (9.8)
	Unknown	21 (19.4)	19 (28.4)	2 (4.9)
Cataract	Yes	74 (68.5)	39 (58.2)	35 (85.4)
	No	18 (16.7)	18 (26.9)	0 (0.0)
	Unknown	16 (14.8)	10 (14.9)	6 (14.6)
Hearing impairment	Yes	7 (6.5)	3 (4.5)	4 (9.8)
	No	17 (15.7)	17 (25.4)	0 (0.0)
	Unknown	84 (77.8)	47 (70.2)	37 (90.2)
Developmental delay	Yes	34 (31.5)	13 (19.4)	21 (51.2)
	No	27 (25.0)	27 (40.3)	0 (0.0)
	Unknown	47 (43.5)	27 (40.3)	20 (48.8)
**Neonatal symptoms**				
Purpura	Yes	18 (16.7)	18 (26.9)	0 (0.0)
	No	48 (44.4)	48 (71.6)	0 (0.0)
	Unknown	42 (38.9)	1 (1.5)	41 (100.0)
Hepatosplenomegaly	Yes	24 (22.2)	24 (35.8)	0 (0.0)
	No	42 (38.9)	42 (62.7)	0 (0.0)
	Unknown	42 (38.9)	1 (1.5)	41 (100.0)
Jaundice	Yes	20 (18.5)	20 (29.9)	0 (0.0)
	No	42 (38.9)	42 (62.7)	0 (0.0)
	Unknown	46 (42.6)	5 (7.5)	41 (100.0)
Suspected meningoencephalitis	Yes	14 (13.0)	14 (20.9)	0 (0.0)
	No	50 (46.3)	50 (74.6)	0 (0.0)
	Unknown	44 (40.7)	3 (4.5)	41 (100.0)
Neonatal thrombocytopenia	n = 41	n = 41	n = 0	
	<150 × 10^9^/liter	26 (63.4)	26 (63.4)	NE
	<50 × 10^9^/liter	11 (26.8)	11 (26.8)	NE
**Serological test for Rubella**				
Rubella specific immunoglobulin M positive		47 (68.1) (n = 69)	46 (71.9) (n = 64)	1 (20.0) (n = 5)
Rubella specific immunoglobulin G positive		14 (100.0) (n = 14)	9 (100.0) (n = 9)	5 (100.0) (n = 5)
**Echocardiography**		n = 106	n = 66	n = 40
Patent ductus arteriosus		92 (86.8)	52 (78.8)	40 (100.0)
Atrial septal defect (patent foramen ovale)		53 (50.0)	48 (72.7)	5 (12.5)
Ventricular septal defect		9 (8.5)	8 (12.1)	1 (2.5)
Atrioventricular septal defect		1 (1.0)	1 (1.5)	0 (0.0)
Pulmonary hypertension by echo		46 (43.4)	33 (50.0)	13 (32.5.0)
Coarctation of aorta		4 (3.8)	4 (6.1)	0 (0.0)
Aortic stenosis		15 (14.2)	4 (6.1)	11 (27.5)
Aortic regurgitation		7 (6.6)	2 (3.0)	5 (12.5)
Pulmonary stenosis		24 (22.6)	10 (15.2)	14 (35.0)
Pulmonary regurgitation		16 (15.1)	9 (13.6)	7 (17.5)
Mitral regurgitation		27 (25.5)	19 (28.8)	8 (20.0)
Tricuspid regurgitation		69 (65.1)	51 (77.3)	18 (45.0)
**Status at discharge**				
Alive		90 (83.3)	50 (74.6)	40 (97.6)
Died/went home to die		18 (16.7)	17 (25.4)	1 (2.4)

CRS; congenital rubella syndrome, IQR; interquartile range, NE; not examined.

^a^Fast breathing, failure to thrive, and/or poor suckling.

### Demographics and symptoms of CRS-PDA and non-CRS-PDA

**Figure 2:**
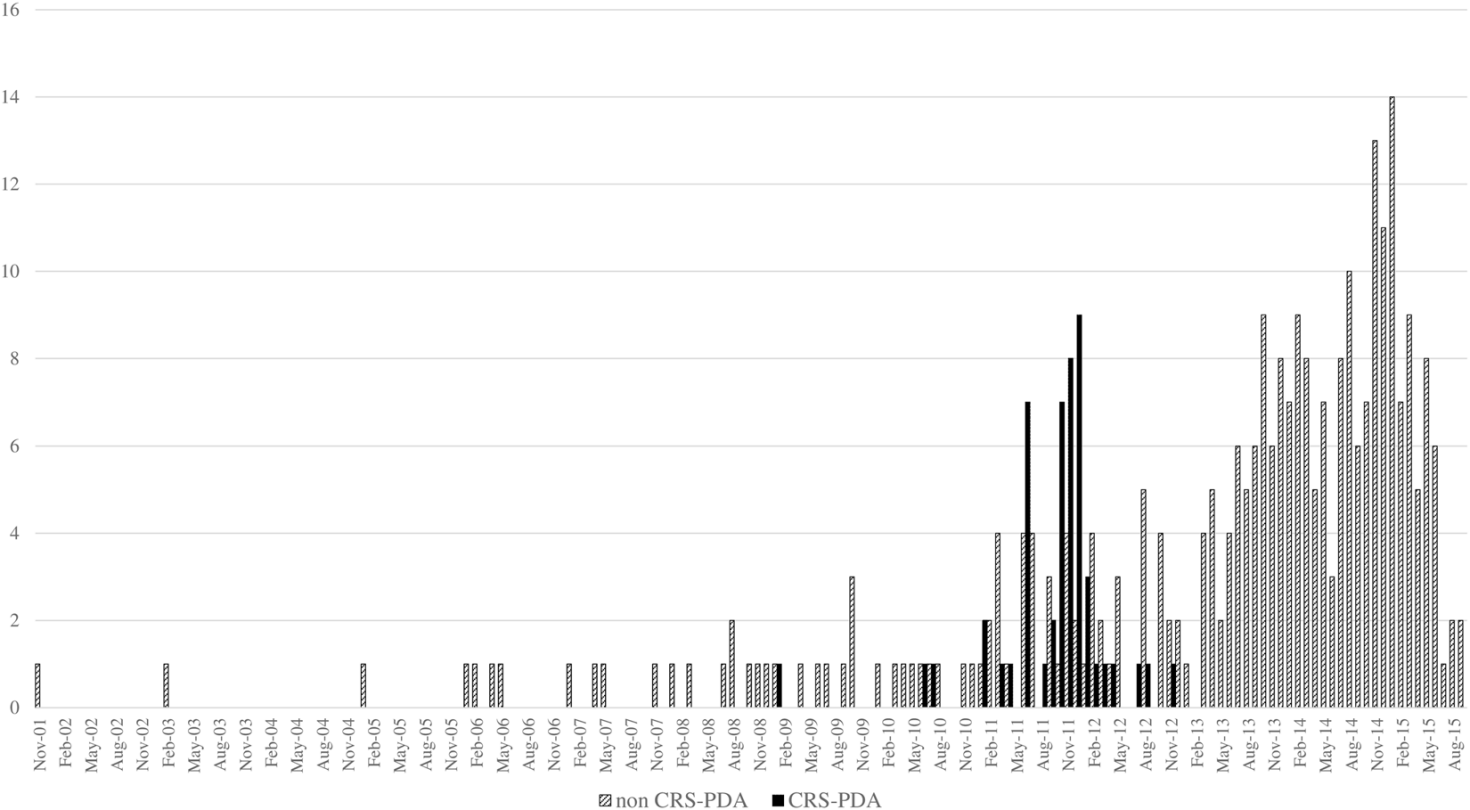
Birth months of the children enrolled in this study who received the transcatheter patent ductus arteriosus occlusion therapy with or without congenital rubella syndrome. CRS-PDA; children with congenital rubella syndrome and patent ductus arteriosus treated by transcatheter closure, non-CRS-PDA; children without congenital rubella syndrome and with patent ductus arteriosus treated by transcatheter closure.

Three-hundred-and-forty patients with transcatheter-occluded PDA, including 50 with CRS (CRS-PDA) and 290 without (non-CRS-PDA), were enrolled (Fig. 1). The former were born between January 2009 and November 2012, with a peak in October 2011. The latter were born mostly between March 2013 and September 2015, with a peak in January 2015, but some were born intermittently from February 2003 to December 2012, overlapping the period when babies with CRS were born (Fig. 2). Children with CRS-PDA were younger (p = 0.06), lighter (p < 0.001), and shorter (p = 0.0026) at the time of transcatheter occlusion therapy and lighter at birth (p < 0.001) than were those with non-CRS-PDA, even though the proportions of preterm births were similar between the groups. Children with CRS-PDA had cardiac defect symptoms, including fast breathing, failure to thrive, and/or poor suckling, more frequently than did children with non-CRS-PDA (82% vs. 25%, p < 0.001) (Table 2). Non-CRS-PDA group included seven children with Down syndrome.

**Table 2:** Characteristics of children who underwent transcatheter PDA occlusion therapy and comparison between PDA with and without CRS.

Characteristics		PDA cases with CRS	PDA cases without CRS	p-value
		Number (%) or median (IQR)	Number (%) or median (IQR)	
		n = 50	n = 290	
**Demographics**				
Sex (boy)		21 (42.0)	94 (32.4)	0.186^b^
Date of birth (range)		10 Jan 2009 - 05 Nov 2012	21 Nov 2001 - 23 Sep 2015	
Age at catheterization (month)		8.4 (6.2–17.5)	11.3 (6.1–34.2)	0.0569^c^
Body weight at catheterization (kilogram)		5.2 (4.7–7.0)	8.0 (6.0–12.0)	<0.001^c^
Body height at catheterization (centimeter)		65 (62–73) (n = 43)	72 (64–90) (n = 282)	0.0026^c^
Body surface area at catheterization (square meter)		0.31 (0.29–0.38) (n = 43)	0.40 (0.33–0.55) (n = 282)	<0.001^c^
**Perinatal information**				
Birthweight and low birthweight		n = 44	n = 269	
Birthweight (gram)		2200 (2000–2600)	2900 (2600–3200)	<0.001^c^
Low birthweight (<2500 gram)		28 (63.6)	49 (18.2)	<0.001^b^
Gestational weeks at birth		n = 50	n = 290	
	37 or more	35 (70.0)	229 (79.0)	0.241^b^
	less than 37	10 (20.0)	47 (16.2)	
	Unknown	5 (10.0)	14 (4.8)	
**Symptoms**				
Symptoms from cardiac disease^f^	Yes	41 (82.0)	71 (24.5)	<0.001^d^
	No	6 (12.0)	216 (74.5)	
	Unknown	3 (6.0)	3 (1.0)	
Cataract	Yes	41 (82.0)	0 (0.0)	<0.001^d^
	No	3 (6.0)	287 (99.0)	
	Unknown	6 (12.0)	3 (1.0)	
Hearing impairment	Yes	7 (14.0)	0 (0.0)	<0.001^d^
	No	4 (8.0)	282 (97.2)	
	Unknown	39 (78.0)	8 (2.8)	
Developmental delay	Yes	27 (54.0)	6 (2.1)	<0.001^d^
	No	3 (6.0)	276 (95.2)	
	Unknown	20 (40.0)	8 (2.8)	
**Echocardiography**		n = 50	n = 287	
Atrial septal defect (patent foramen ovale)		7 (14.0)	28 (9.8)	0.449^d^
Ventricular septal defect		1 (2.0)	7 (2.4)	>0.999^d^
Pulmonary hypertension by echo		17 (34.0)	71 (24.7)	0.164^b^
Coarctation of aorta		0 (0.0)	2 (0.8)	>0.999^d^
Aortic stenosis		11 (22.0)	5 (1.7)	<0.001^d^
Aortic regurgitation		5 (10.0)	12 (4.2)	0.150^d^
Pulmonary stenosis		17 (34.0)	2 (0.7)	<0.001^d^
Pulmonary regurgitation		8 (16.0)	21 (7.3)	0.055^d^
Mitral regurgitation		9 (18.0)	118 (41.1)	0.002^d^
Tricuspid regurgitation		24 (48.0)	147 (51.2)	0.674^b^
**Angiography**				
		n = 47	n = 276	
Systolic pressure of aorta (mmHg)		90 (75–116)	74 (64–83)	<0.001^c^
Mean pressure of aorta (mmHg)		69 (56–79)	53 (46–61)	<0.001^c^
Diastolic pressure of aorta (mmHg)		50.5 (38–62) (n = 46)	36 (30–42.5)	<0.001^c^
		n = 39	n = 235	
Systolic pressure of main PA (mmHg)		49 (30–67)	33 (26–43)	<0.001^c^
Mean pressure of main PA (mmHg)		36.5 (25–47) (n = 38)	25 (19–32)	<0.001^c^
Diastolic pressure of main PA (mmHg)		27 (17–37)	17 (13–24) (n = 234)	<0.001^c^
PH (mean pressure of main PA ≧25 mmHg)		29 (76.3) (n = 38)	120 (51.1)	0.005^d^
PDA type		n = 44	n = 285	
A (conical)		33 (75.0)	244 (85.6)	0.002^e^
B (window)		0 (0.0)	2 (0.7)	
C (tubular)		7 (15.9)	9 (3.2)	
D (complex)		0 (0.0)	1 (0.4)	
E (elongated)		4 (9.1)	29 (10.2)	
PDA size		n = 44	n = 282	
Aorta side diameter (mm)		8.1 (6.8–9.8)	9.0 (7.7–10.8)	0.0836^c^
PA side diameter (mm)		2.7 (1.5–4.1)	1.9 (1.4–2.7)	0.0074^c^
Length (mm)		8.0 (6.8–9.6)	6.8 (5.6–8.7)	0.0019^c^
Ratio of PA side diameter to aorta side diameter		0.3 (0.2–0.4)	0.2 (0.2–0.3)	0.0015^c^
Aorta diameter		n = 43	n = 280	
Diameter proximal to PDA (mm)		6.8 (5.1–7.9)	7.7 (6.2–9.5)	0.0026^c^
Diameter distal to PDA (mm)		7.6 (6.8–8.7)	8.6 (7.3–9.9)	0.0031^c^
Estimated aorta diameter^a^		7.4 (7.0–8.4) (n = 43)	9.6 (7.7–11.0) (n = 282)	<0.001^c^
Aorta diameter proximal to PDA/estimated aorta diameter^a^ (%)		93.2 (69.1–105.1) (n = 39)	83.6 (75.1–94.3) (n = 274)	0.2686^c^
**Status at discharge**				
Alive		50 (100.0)	287 (99.0)	>0.999^d^
Died/went home to die		0 (0.0)	3 (1.0)	

PDA; patent ductus arteriosus, CRS; congenital rubella syndrome, SD; standard deviation, IQR; interquartile range, PA; pulmonary artery, ADO-I; PDA occluders with retention skirt, ADO-II; Amplatzer™ Duct Occluder II.

^a^Estimated diameter of descending aorta just distal to left subclavian artery.

^b^Chi square test, ^c^Wilcoxon rank sum test, ^d^Fisher’s exact test.

^e^Frequency of tubular-type PDA was compared by Fisher’s exact test.

^f^Fast breathing, failure to thrive, and/or poor suckling.

### Characteristics of cardiac defects identified by echocardiography and angiography

Using echocardiography, we found that mitral regurgitation occurred among children with CRS-PDA (18%) less frequently than in those with non-CRS-PDA (41%), but children with CRS-PDA had pulmonary (34%) and aortic stenosis (22%) more frequently than did children with non-CRS-PDA (0.7% and 1.7%, respectively) (Table 2). Among the 17 cases of pulmonary stenosis in those with CRS-PDA, eight were valvular stenosis alone, five were valvular and supravalvular stenosis, three were supravalvular stenosis alone, and one was valvular and left peripheral stenosis. Per the pressure gradient measurement of pulmonary stenosis, two cases were mild (<36 mmHg), 12 were moderate (36–64 mmHg), and three were severe (>64 mmHg)^7^. Eleven cases in the CRS-PDA group had aortic stenosis: six were mild (mean gradient < 20 mmHg), two were moderate (20–39 mmHg), and three were severe (≥40 mmHg)^8^.

PH was detected during cardiac catheterization (mean pulmonary artery pressure [mPAP] ≥ 25 mmHg) more frequently in CRS-PDA (76%) than in non-CRS-PDA (51%) (p = 0.005). The aortic and main PA pressures in systole and diastole were higher in those with CRS than in those without, although the former patients were younger than the latter (Table 2).

**Figure 3:**
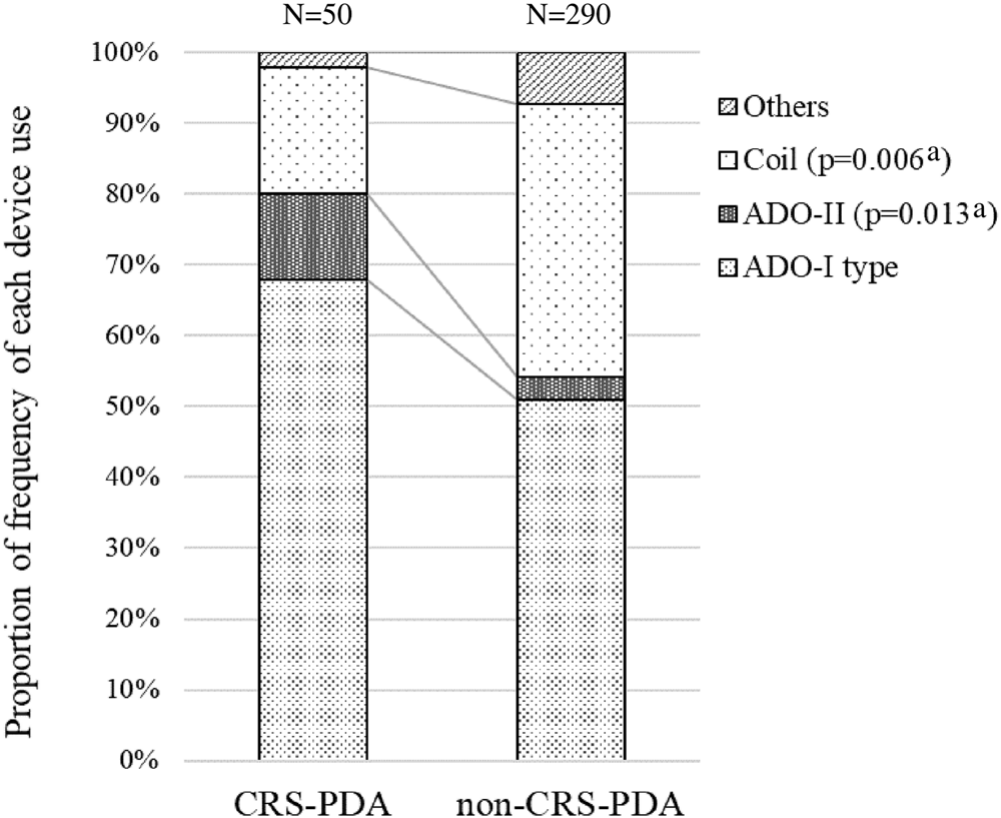
Proportion of frequency of each device use for patent ductus arteriosus occlusion in CRS-PDA and non-CRS-PDA. (**a**) Fischer’s exact test CRS-PDA; children with congenital rubella syndrome and patent ductus arteriosus treated by transcatheter closure, non-CRS-PDA; children without congenital rubella syndrome and with patent ductus arteriosus treated by transcatheter closure, ADO-I type; PDA occluders with retention skirt, ADO-II; Amplatzer™ Duct Occluder II, Others; other occluders including muscular ventricular septal defect occluder, atrial septal defect occluder, coil for ventricular septal defect, and use ADO-II and coil in combination.

**Figure 4:**
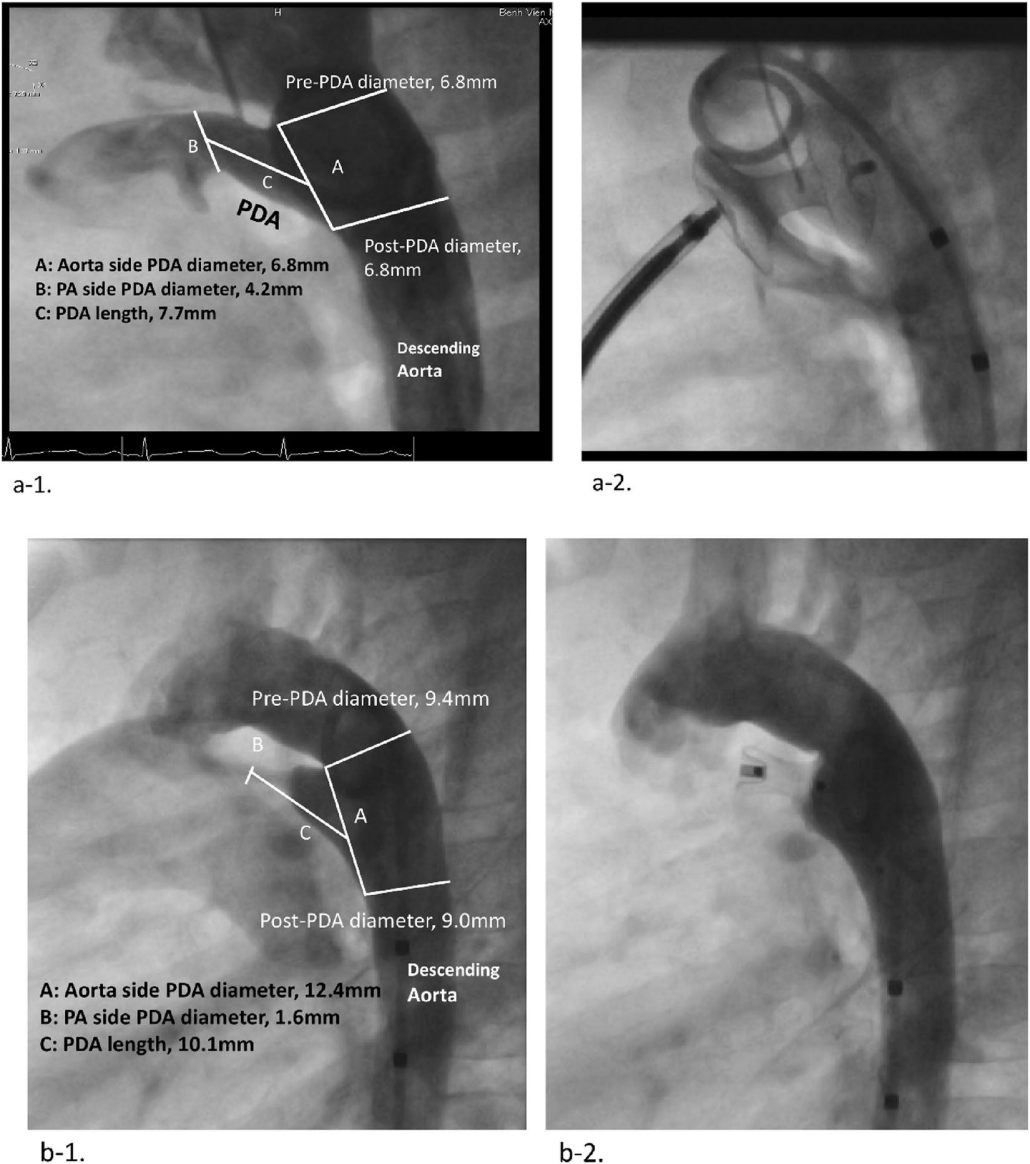
Type C PDA with CRS and type A PDA without CRS. (**a**) Type C PDA of a 24-month-old girl with CRS (a-1) and the closure with a muscular VSD occluder (a-2). (a) Type A PDA of 29-month-old girl (b-1) and the closure with Amplatzer™ Duct Occluder (b-1). PDA; patent ductus arteriosus, CRS; congenital rubella syndrome, VSD; ventricular septal defect.

The proportion of tubular-type PDA was higher among patients with CRS-PDA (16%) than in those with non-CRS-PDA (3%) (p = 0.020) (Table 2). The diameter on the pulmonary artery (PA) side and PDA length were significantly larger and longer in the CRS-PDA than in non-CRS-PDA (p = 0.0074 and p = 0.0019, respectively). The ratio of the PA side diameter to the aorta side diameter was larger in the CRS-PDA than in non-CRS-PDA (p = 0.0015, Table 2). In terms of PDA closure devices, a coil occluder was used more frequently in those without CRS (p = 0.006), and Amplatzer™ Duct Occluder II (ADO-II) was used more frequently in those with CRS (p = 0.013) (Fig. 3). Figure 4 shows angiograms of tubular-type PDA with CRS occluded by a double-disc device and conical-type PDA without CRS occluded by an Amplatzer™ Duct Occluder (ADO-I) type device.

The aorta diameters, both proximal and distal to the PDA, were much smaller in the CRS-PDA than in non-CRS-PDA (p = 0.0024 and p = 0.0061, respectively); however, the ratios of the aorta diameter proximal to the PDA to that just distal to the left subclavian artery that were estimated with the body surface areas were similar between the groups (p = 0.2686).

## Discussion

This is the first study to investigate the morphological and hemodynamic characteristics of PDA in children with CRS and to compare them with those in children with PDA without CRS. This study demonstrated that tubular-type PDA is more frequently observed in children with CRS.

### Epidemiological characteristics of children who had therapy for PDA

We found that the number of transcatheter PDA occlusions in a hospital by birth date peaked in October and November 2011, similar to the number of infants with CRS born in Vietnam^3,4^. The birth years of children who underwent PDA ligation surgery also peaked in 2011, although the number was much less than that of transcatheter occlusion procedures at the hospital. We believe that the excessive morbidity of those with PDA was due to CRS, and that the cardiovascular burden of CRS should have been much larger than that described in this study.

### Clinical characteristics of children with CRS

While the proportions of children with CRS in this study who had low birthweights and those who had cataracts were similar to those seen in other studies^4,9,10^, hearing impairment and developmental delay were detected much less frequently than in other reports^9–12^. In Vietnam, there is not routine active screening program for hearing impairment or developmental delay in general and the enrolled cases did not have such examinations during this hospitalization for cardiac catheterization, so mostly lacked those information in the medical charts. Although neonatal findings suggestive of CRS, such as purpura, hepatosplenomegaly, jaundice, and thrombocytopenia, were also less prevalent, we presume that they were simply unrecorded in this hospitalization.

Cardiac defects were disproportionally frequent among children in this study because many of them were identified in the Department of Cardiology. PDA was the most prevalent cardiac defect in this (the middle column in Table 1) and in previous studies^4,13^. The frequency of atrial septal defect/patent foramen ovale was disproportionally higher than those in previous studies^4,13^, probably because it included many cases of patent foramen ovale in young patients^4,14^. Pulmonary hypertension, which was significantly associated with mortality in CRS in our previous study^4^, was also frequently observed in this study.

### Cardiac characteristics in CRS-PDA comparing with non-CRS-PDA

Children with CRS were smaller in body size at the time of catheterization and at birth than the non-CRS group, even though the proportion of preterm births was similar. The higher proportion of girls (67.6%) with non-CRS-PDA is consistent with that in a previous study reporting that the ratio of female to male patients was approximately 2:1^15^. Conversely, the incidence of CRS-PDA was less female-dominated in this study.

Pulmonary and aortic stenosis were detected with echocardiography much more frequently for CRS-PDA than for non-CRS-PDA. Previous studies also demonstrated pulmonary stenosis to be a common finding of CRS^4,12,15^. Most cases of pulmonary stenosis with CRS-PDA in this study were valvular, supravalvular, or combined; peripheral PA stenosis was rare. This was different from a review of a series of CRS studies with catheterization data by Oster *et al*.^16^, which showed that 73% and 16% of patients with CRS had branch PA and pulmonary valve stenosis, respectively. This disparity may be due to the limitations of echocardiography, which may not image distal PA stenosis reliably^16^, especially when combined with proximal PA stenosis whose color Doppler jet can overlap with the distal one. We observed that aortic stenosis is a characteristic finding in CRS. Stuckey found aortic stenosis in one patient and aortic coarctation with aortic stenosis in another among 44 children with a history of maternal rubella during pregnancy^17^. The present study demonstrated that the aorta diameters, both proximal and distal to the PDA, were much smaller in children with CRS-PDA than in those without CRS; however, the difference disappeared after we adjusted for the body surface area. Hastreiter *et al*. reported hypoplasia of the aortic isthmus in 16% of infants with CRS^13^. In this study, the findings in the PA and aorta may suggest that both were poorly developed in patients with CRS and were accompanied by poor body size growth and/or arterial narrowing due to intimal proliferation as discussed below^18^.

Mitral regurgitation was less prevalent in CRS-PDA than in non-CRS-PDA, possibly because combined progressive PH reduced left heart volume load due to high pulmonary resistance^19^. Qp/Qs should have been assessed to confirm this but it was not recorded in the medical charts except for in two cases.

Direct blood pressure measurement during catheterization allowed us to detect not only higher PA pressure but also higher aortic pressure in patients with CRS-PDA, even though they had a younger median age than those without CRS. Esterly and Oppenheimer reported vascular lesions in 13 autopsied infants with CRS and found ostial stenosis of the renal artery with intimal fibromuscular proliferation in an infant with systemic hypertension^18^. Hypertension is one of the first manifestations in adults with CRS^20^. A study showed that fetuses and infants with intrauterine growth restriction had significant aortic intima-media thickening and higher systolic blood pressure than those who were gestational-age appropriate^21^. In this study, the increased diastolic blood pressure in patients with CRS might indicate increased peripheral vascular resistance due to narrowing of the systemic arteries, possibly including the renal artery. Our finding suggests that many children with CRS could have hypertension developing in infancy that would become apparent when the arteries narrow with age^20^.

### Types and sizes of PDA and device types in CRS-PDA comparing with non-CRS-PDA

In patients with CRS-PDA, the proportion of tubular-type (type C) PDA was higher, the PA side diameter was larger, and PDA was longer than in those with non-CRS-PDA. PH occurred more frequently in CRS-PDA and patients developed symptoms due to PH or heart failure at earlier ages. Accordingly, they needed transcatheter PDA occlusion therapy at younger age and with a smaller body size, and the ADO-II occlusion device was used more frequently.

Percutaneous closure of type C PDA is challenged by difficulty in satisfactorily stabilizing the prosthesis due to lack of a sufficient ampulla^5,6^. The ADO-I devices, which have an asymmetric design that allows its skirt to be positioned within the aortic ampulla, leads to the risk of displacement or embolization in the aorta^22,23^ in type C PDA, especially with high PA pressure, which is frequently seen in those with CRS. Symmetrically designed devices such as the ADO-II^24^, which has two retention discs that are positioned on both sides of the duct, or occluders for atrial septal defects or muscular ventricular septal defects^25^, are usually used in the hospital for type C PDA to avoid this complication. However, the device can protrude into the aorta or left PA, especially in small children^6,26^. This risk is inherently higher in patients with CRS and those with a smaller body size, smaller aorta, and more severe stenosis in the aorta or PA. PDA coils for a small PDA (PA side diameter < 4 mm) and ventricular septal defect coils for large PDA (PA side diameter of ≥4 mm) are options to close type C PDAs in patients with small aortas.

Histologically, smooth muscle cells migrate into the subendothelial region to form intimal thickening, leading to ductus closure^27^. PDA tissue derived from patients with CRS resembles that of a very immature ductus and exhibits an extensive subendothelial elastic lamina in the ductus wall and poorly formed intimal thickening^27,28^. No previous study has carefully addressed the issue of histological differences among the types of PDA; however, based on the morphological similarity (tubular shape) of CRS-PDA to the ductus arteriosus seen in fetuses, we speculate that the intimal thickening process is halted in the early fetal phase by rubella virus infection^28^.

### Limitations

This study has some potential limitations. Some CRS-PDA cases may have been included in the non-CRS-PDA group. Since this was a retrospective study in which medical charts and cardiologic data were reviewed, we were unable to distinguish if patients actually lacked clinical manifestations of CRS, if these manifestations were not recorded, or if they are still pending (e.g., hearing impairment or developmental delay). However, we believe we could minimize this by excluding children with non-cardiac CRS symptoms without known underlying diseases that can cause these symptoms, from the non-CRS-PDA group.

## Conclusion

In conclusion, tubular-type PDA was frequently seen in patients with CRS and accompanied by pulmonary/systemic hypertension and pulmonary/aortic stenosis. Transcatheter closure of CRS-PDA requires a more cautious choice of device and more detailed follow-up after the intervention.

## Methods

### Study location and participants

The study was conducted at CH1, Ho Chi Minh City, which covers sick children living in the southern and central areas of Vietnam. CH1 established the Department of Cardiology and started cardiac catheterization including transcatheter PDA therapy in 2009. For the first part of the study to describe clinical manifestations of CRS, we retrospectively enrolled patients with confirmed or probable CRS who were admitted to the Department of Cardiology or Neonatology between December 2010 and December 2012 and actively screened for CRS in the previous study^3^, and those with probable CRS and PDA hospitalized between December 2009 and December 2015 who were identified from the department’s patient list. For the second part of the study to investigate morphological and hemodynamic characteristics of PDA in children with CRS, we targeted children with CRS who had PDA catheterization therapy in this hospital and enrolled to the first part of the study (CRS-PDA) and additionally enrolled those who underwent transcatheter PDA closure between July 2014 and December 2015 (non-CRS-endemic period) and had no other CRS symptoms through a scan of the catheterization logbook; these were regarded as non-CRS (non-CRS-PDA). Children who had transcatheter PDA closure in this period and showed other symptoms suspected of CRS were regarded as non-CRS if they had another etiology to reasonably explain those symptoms (e.g., PDA with cataract in Down syndrome was regarded as non-CRS-PDA). The enrollment flow is shown in Fig. 1. CRS was diagnosed using the modified definition of the Centers for Disease Control guideline (Fig. 1 and Supplemental Table 2)^29^. In this hospital, patients with PDA undergo either transcatheter PDA closure or surgical PDA ligation when they have left atrial and/or left ventricular enlargement, PH, or net left-to-right shunting^30^. Surgical ligation is selected for small babies (body weight < 3 kg), cases with failure to transcatheter closure due to technical issue, those with huge PDA (PA side diameter > 12 mm), or those with transcatheter closure complication (e.g. device embolization). Only 2.7% of closure of PDA is surgical in this hospital.

### Data collection and study design

We reviewed the charts and angiographic images of those who underwent transcatheter PDA closure and collected demographic and clinical information and ultrasonographic and angiographic results using a standardized data collection form. Two experienced pediatric cardiologists independently reviewed the angiographic images with Syngo FastView® (Siemens Healthineers, Erlangen, Germany), determined the PDA types, and measured the PDA and aorta sizes. They resolved any disagreements by discussion. We also reviewed the department records from 2011 to 2015 to determine the change in yearly numbers of catheterization for PDA occlusion. We described the characteristics of the children with CRS-PDA and compared them with those with non-CRS-PDA. The primary outcome was the proportion of type C (tubular-type) PDA, and the secondary outcomes were other cardiac comorbidities, PDA diameters (both PA and aortic sides), aorta diameter, PA and aorta pressure, and type of PDA occlusion device.

### PDA type and device for PDA occlusion

We used Krichenko’s angiographic classification of PDAs, using the narrowest end of the ductus as a landmark^5^. In type A (conical), the narrowest segment is at the pulmonary insertion, with a well-defined ampulla at the aortic end; in type B, the ductus is short and narrowed at the aortic insertion. Type C (tubular) comprises the tubular ductus without constriction. In type D, the ductus has multiple constrictions. Type E has an elongated, conical appearance, and the constriction is remote from the anterior border of the trachea. We categorized the PDA occluders with retention skirts as the ADO-I type. This includes the Amplatzer™ Duct Occluder (ADO-I, St. Jude Medical, St. Paul, MN), Occlutech® PDA Occluder (Occlutech, Helsingborg, Sweden), Nit-Occlud® PDA-R (PFM Medical AG, Cologne, Germany), and Cocoon Duct Occluder (Vascular Innovations, Nonthaburi, Thailand). Other types were the Amplatzer™ Duct Occluder II (ADO-II, St. Jude Medical, St. Paul, MN), which has dual articulating discs, a coil, which is a simple device to occlude a small PDA, an atrial septal defect occluder, muscular ventricular septal defect occluder, coil for ventricular septal defect, and a combination of two devices. In this hospital, device for PDA occlusion is selected following the patient’s PDA type, size, the aorta size, and the body weight, e.g., ADO-I type is used for most cases with type A PDA, ADO-II is used for small (PA side diameter of PDA < 4 mm) type E, D, and A PDA with enough space in aorta (diameter of descending aorta > 10 mm), and PDA coil is used for small (PA side diameter of PDA < 4 mm) type A PDA with small descending aorta.

### PH and symptoms of cardiac disease

PH, defined as systolic pulmonary artery pressure (sPAP) ≥ 35 mmHg^31^ or mPAP ≥ 25 mmHg^32^, was detected based on invasive measurement of mPAP during catheterization, an increased right ventricular pressure inferred from the tricuspid regurgitant velocity without the presence of pulmonary valve stenosis or outflow tract obstruction (estimated sPAP), or an increased PAP inferred from the pulmonary regurgitation velocity (estimated mPAP) with echocardiography. sPAP and mPAP were estimated with echocardiography using the simplified Bernoulli equation: PAPs (mmHg) = 4 × (tricuspid regurgitation velocity [meter/second])^2^ + (mean right atrial pressure [mmHg])^33^, mPAP (mmHg) = 4 × (maximum diastolic pulmonary regurgitation velocity [meter/second])^2^ + (mean right atrial pressure [mmHg])^33^, assuming the mean right atrial pressure is 10 mmHg. Symptoms included poor sucking, failure to thrive, fatigue, tachypnea, recurrent pneumonia, and cyanosis.

### Statistical analysis

Chi-square or Fisher’s exact test (for categorical variables) and Wilcoxon rank sum test (for continuous variables) were used to compare demographic, clinical, or cardiac characteristics, including primary and secondary outcomes, between the two groups. Continuous variables were expressed as median and interquartile range. The ratio of the aorta diameter proximal to the PDA to the estimated diameter of the aorta just distal to the left subclavian artery’s starting point was calculated and compared between the two groups to adjust the aorta’s diameter by body size: diameter of the descending aorta just distal to the left subclavian artery (mm) = 15.3 × (body surface area in m^2^) + 2.6^34^. Body surface area was calculated using Haycock’s formula:^35^ body surface area (m^2^) = 0.024265 × height (cm)^0.3964^ × weight (kilogram)^0.5378^. P-values < 0.05 were considered statistically significant. The statistical analyses were conducted using STATA version 14.0 (StataCorp, College Station, TX).

### Ethics

The patients’ charts were reviewed by a doctor in the Department of Cardiology of CH1, and the data were anonymized. The institutional review boards of CH1, Ho Chi Minh City, and the Institute of Tropical Medicine, Nagasaki University, approved this study. This study was conducted in accordance with relevant guidelines and regulations.

## Supplementary information

Supplemental table 1, Supplemental table 2, Supplemental figure
