# Supplementary material for: Characteristics of Patent Ductus Arteriosus in Congenital Rubella Syndrome
Source: Sci Rep. 2019 Nov 19;9:17105. doi: 10.1038/s41598-019-52936-6 (PMC6863812; doi:10.1038/s41598-019-52936-6)

Title page

Characteristics of Patent Ductus Arteriosus in Congenital Rubella Syndrome

Michiko Toizumi, MD, PhD,[1,2] Cam Giang T. Do, MD,[3] Hideki Motomura, MD, PhD,[4] Tin N. Do, MD,[3] Hirofumi Fukunaga, MD[5], Makiko Iijima, MD,[6] Nhan NT. Le, MD,[7] Hung Thanh Nguyen, MD, PhD,[7] Hiroyuki Moriuchi, MD, PhD,[2,8] and Lay-Myint Yoshida, MD, PhD[1,8*]

Affiliations:

[1]Department of Pediatric Infectious Diseases, Institute of Tropical Medicine, Nagasaki University, Nagasaki, Japan; [2]Department of Global Health, School of Tropical Medicine and Global Health, Nagasaki University, Nagasaki, Japan; [3]Department of Cardiology, Children's Hospital 1, Ho Chi Minh City, Vietnam; [4]Department of Pediatrics, Nagasaki Medical Center, Omura, Japan; [5]Department of Pediatrics, Nagasaki University Hospital, Nagasaki, Japan; [6]Expanded Programme on Immunization, WHO representative office in Viet Nam, Hanoi, Vietnam; [7]Outreach and International Department, Children's Hospital 1, Ho Chi Minh City, Vietnam; [8]Graduate School of Biomedical Sciences, Nagasaki University, Nagasaki, Japan

***Corresponding author**

Lay-Myint Yoshida, Department of Pediatric Infectious Diseases, Institute of Tropical Medicine, Nagasaki University, 1-12-4 Sakamoto, Nagasaki 852-8523, Japan. E-mail: lmyoshi@nagasaki-u.ac.jp, Tel.: +81-95-819-7764, Fax: +81-95-819-7844

Supplemental table 1. Numbers of transcatheter therapy and transcatheter patent ductus arteriosus occlusion therapy at the Department of Cardiology, Children's Hospital 1

Year	2011	2012	2013	2014	2015	Total in 5 years
Transcatheter therapy (a)	494	770	553	581	580	2978
Transcatheter PDA occlusion (b)	419	495	273	187	225	1599
(b)/(a) (%)	84.8	64.3	49.4	32.2	38.8	53.7

PDA; patent ductus arteriosus

Supplemental table 2. Definition of Congenital Rubella Syndrome (CRS)[29]

1) Symptoms suggesting CRS are symptoms in Group (A) and (B) as below.

Group A	Group B
congenital heart disease	purpura
cataract(s)	jaundice within 24 hours after birth
glaucoma	hepatosplenomegaly
suspected hearing impairment	meningoencephalitis[a]
	developmental delay
	microcephaly

2) Confirmed CRS and Probable CRS

Confirmed CRS

one with any clinical manifestations of CRS (A and B) confirmed on a laboratory test[b].

Probable CRS

one that is not laboratory confirmed but includes either 2 of the clinical signs listed in group (A) or 1 of the clinical signs listed in group (A) and 1 of the clinical signs listed in group (B) with no evidence of any other etiology.

a; Meningoencephalitis was defined clinically according to the presence of abnormal neurologic findings and signs of meningeal irritation, such as neck stiffness, altered consciousness, bulging fontanels, and convulsions.
b; Cases were laboratory confirmed based on the detection of rubella-specific immunoglobulin M antibodies in infancy.

Supplemental figure. Number of transcatheter patent ductus arteriosus occlusion therapy between 2011 and 2015, at Children's Hospital 1 in Ho Chi Minh City, sorted by birth months

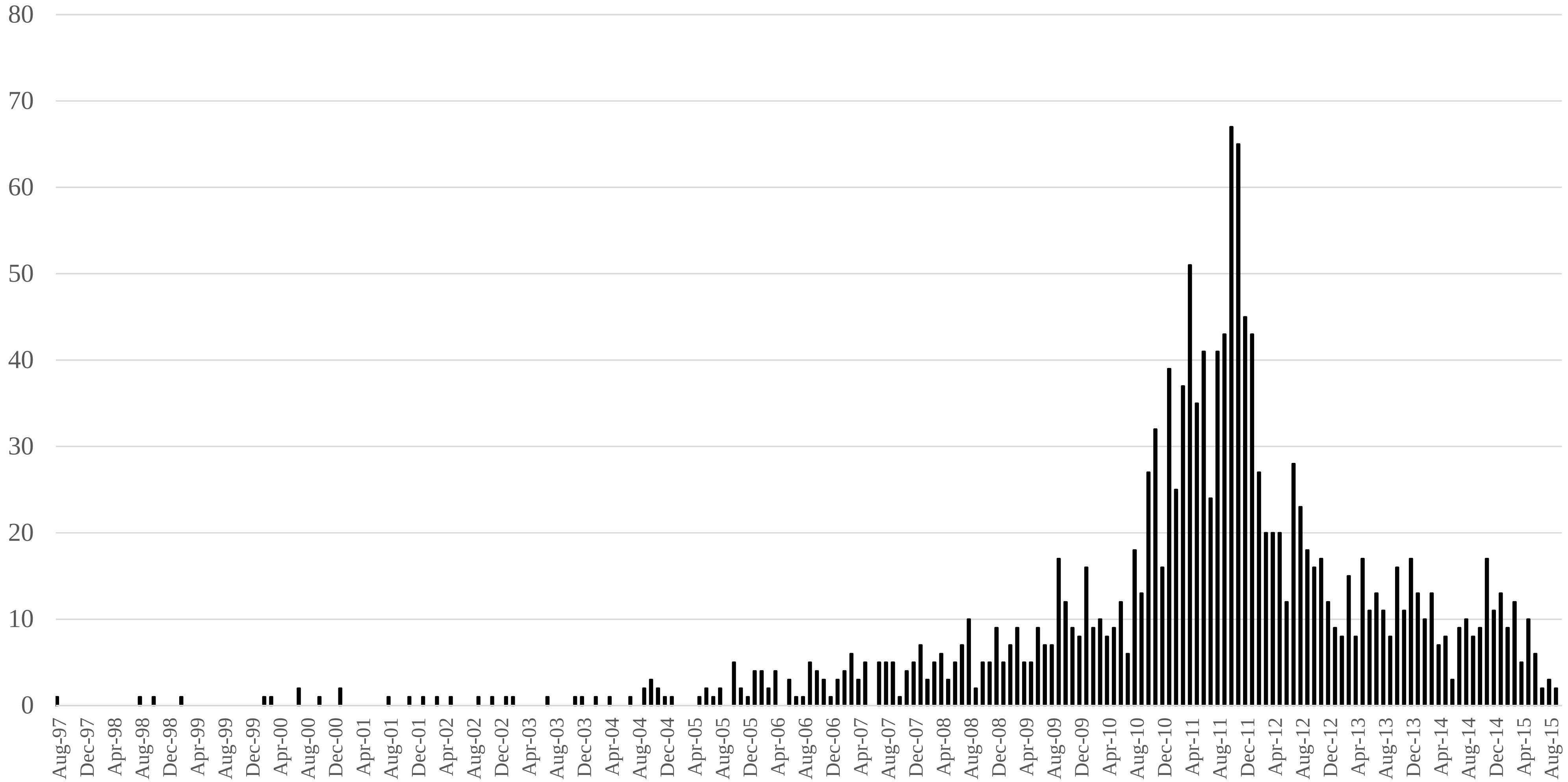

Supplement: Supplementary file 1 — Supplemental table 1, Supplemental table 2, Supplemental figure [file 41598_2019_52936_MOESM1_ESM.pdf]
